# Supplementary material for: Natural Killer Cell-Secreted IFN-γ and TNF-α Mediated Differentiation in Lung Stem-like Tumors, Leading to the Susceptibility of the Tumors to Chemotherapeutic Drugs
Source: Cells. 2025 Jan 10;14(2):90. doi: 10.3390/cells14020090 (PMC11763808; doi:10.3390/cells14020090)

Supplementary data

Figure Legends

Figure S1. Surface markers and NK cell-mediated cytotoxicity in hA549. NK cells were left untreated and treated with IL-2 (1000 U/ml) or a combination of IL-2 (1000 U/ml) and anti-CD16 mAbs (3 µg/ml) overnight before they were used as effectors to measure NK cell cytotoxicity against $^{Cr}51$ labeled hA549 cells. After 4 hours of incubation of NK cells with hA549 the gamma counter counted the radioactivity released in the supernatants and the levels of cytotoxicity were determined using LU 30/10^6. LU 30/10^6 denotes the number of NK cell effectors needed to lyse 30% of hA549 **(A)**. hA549 were stained using PE-conjugated antibodies against isotype control, CD44, CD54, B7H1, and MHC-class I, and the level of surface expressions was determined by flow cytometry analysis **(B)**.

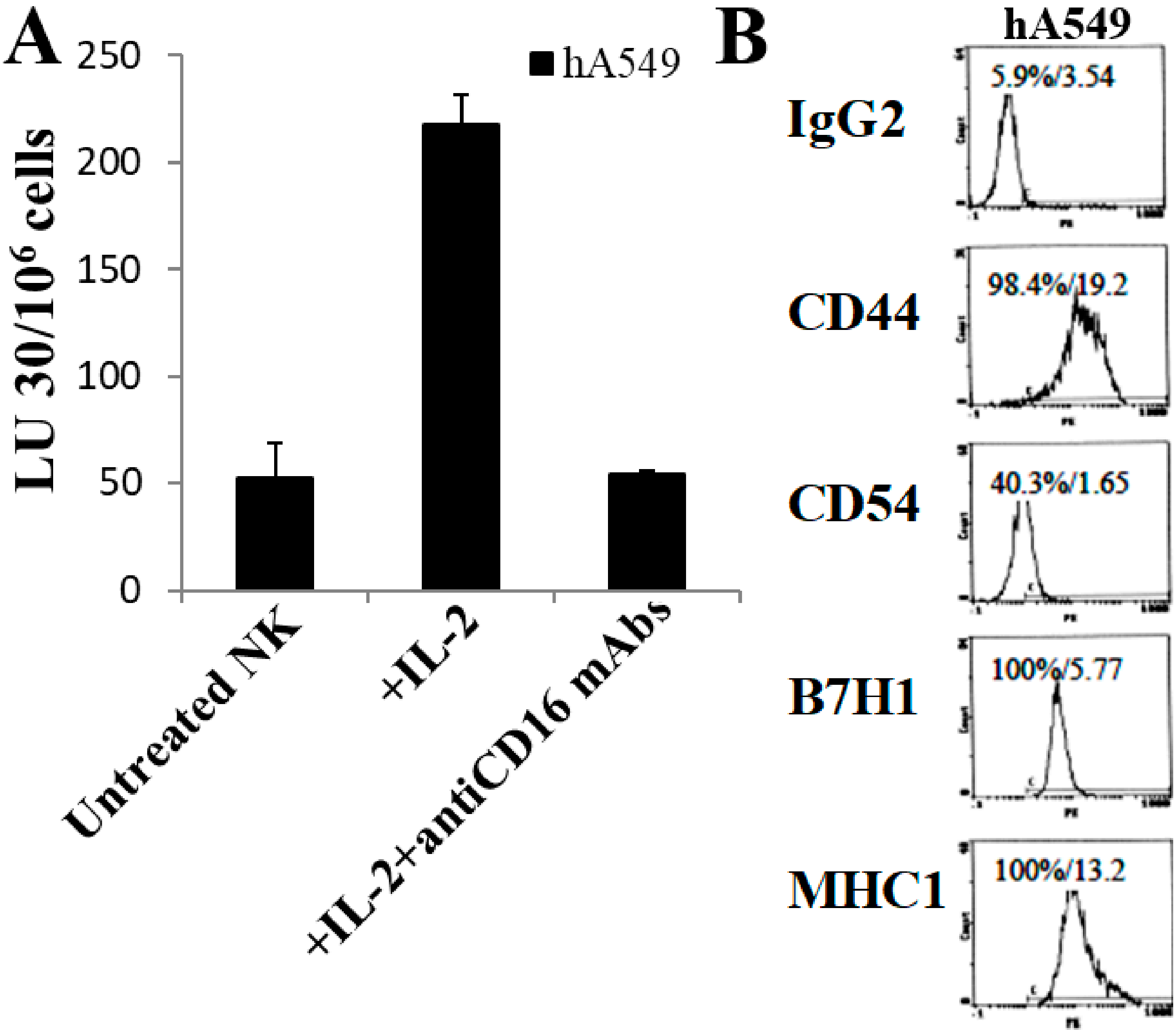

 https://doi.org/10.3390/cells14020090

Figure S2. Treatment of NK cells with IL-2 and anti-CD16 mAbs increases secretion of TNF-α and IFN-γ NK cells were left untreated or treated with IL-2 (1000 U/mL) or treated with IL-2 (1000 U/mL) and anti-CD16 mAbs (3 μg/mL) overnight. Afterward, the supernatants were harvested and tested for TNF-α **(A)** and IFN-γ **(B)** release using ELISA.

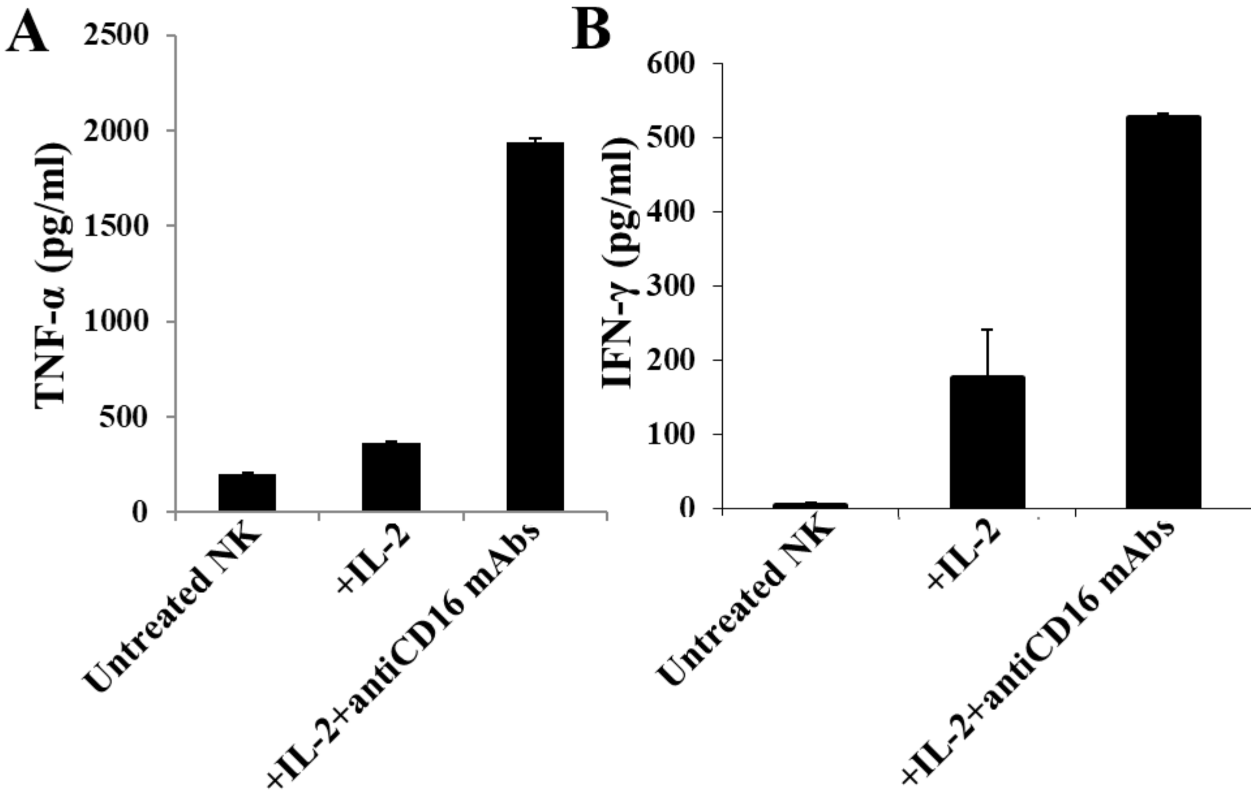

Figure S3. Surface expression levels of hA549 **after treatment with rIFNγ and rTNFα.** hA549 cells were treated with rTNF-α (20 ng/mL) or rIFN-γ (200U/mL) or rTNF-α (20 ng/mL) + rIFN-γ (200 U/mL) for 24 hours. After which, surface expression of CD54, B7H1, and MHC-class I on hA549 were assessed using flow cytometric analysis.

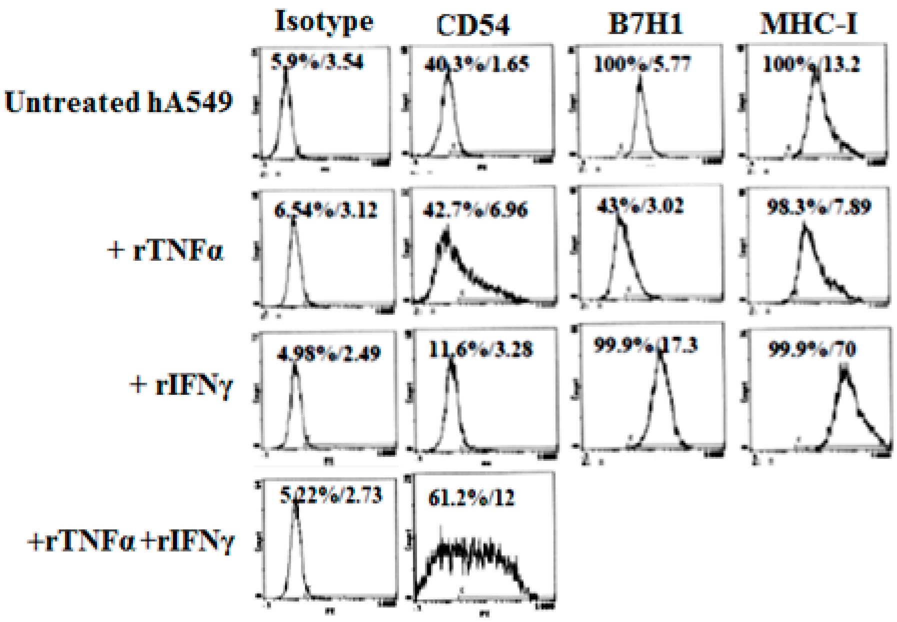

Figure S4. Treatments with anti-IFN-γ and anti-TNF-α blocked NK cell supernatant-induced differentiation of SCAP cells. NK cells were left untreated, or treated with a combination of IL-2 (1000 U/ml) and anti-CD16 mAbs (3μg/mL) overnight before the supernatants were harvested to treat SCAP cells. SCAP cells were treated with supernatants of untreated or IL-2 (1000 U/ml) and anti-CD16 mAbs (3μg/mL) treated NK cells alone or with αTNFα mAbs (1:100) + αIFNγ mAbs (1:100) for six days. SCAP cells were prepared as described in Fig. S1A, after which, surface expression of CD44, MHC-class I, B7H1 and CD54 on SCAP were assessed using flow cytometric analysis **(A)**. SCAP cells were prepared as described in Fig. S2A, after which, the cells were stained with propidium iodine (PI) to determine percent cell death using flow cytometric analysis **(B)**. NK cells were left untreated, treated with IL-2 (1000 U/ml) or treated with a combination of IL-2 (1000 U/ml) and anti-CD16 mAbs (3μg/mL) overnight before they were used as effectors against ^{51}Cr-labeled SCAP cells in 4 hours cytotoxicity assay. Lytic units (LU) 30/10^6 cells were determined using the inverse number of NK cells required to lyse 30% of target cells × 100 **(C)**.

A

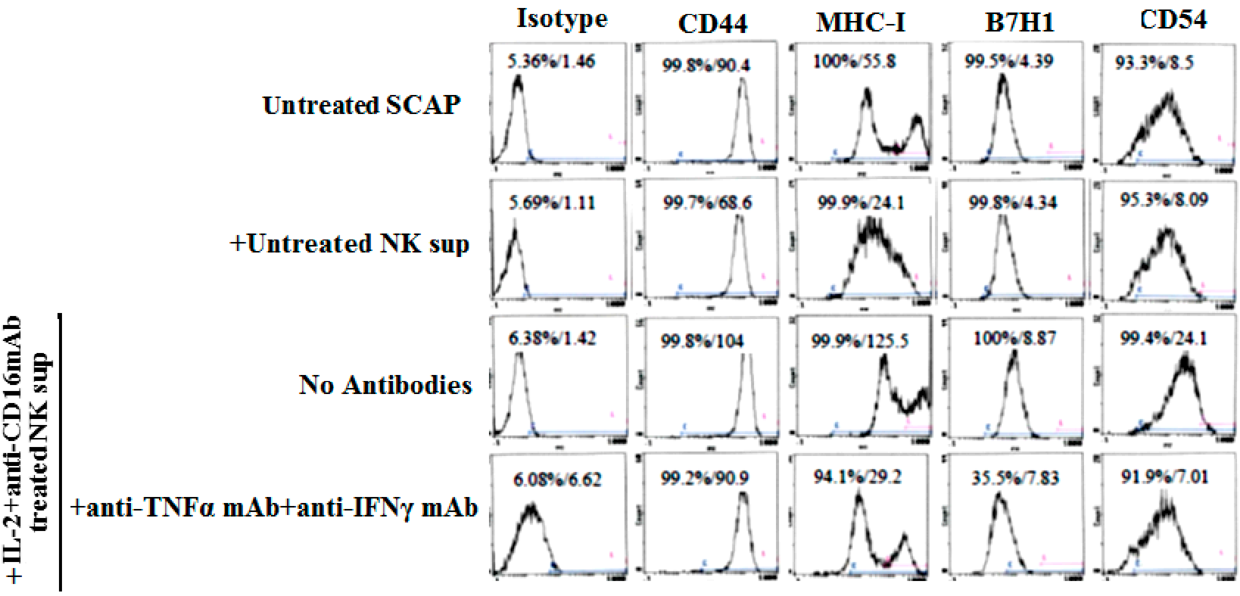

B

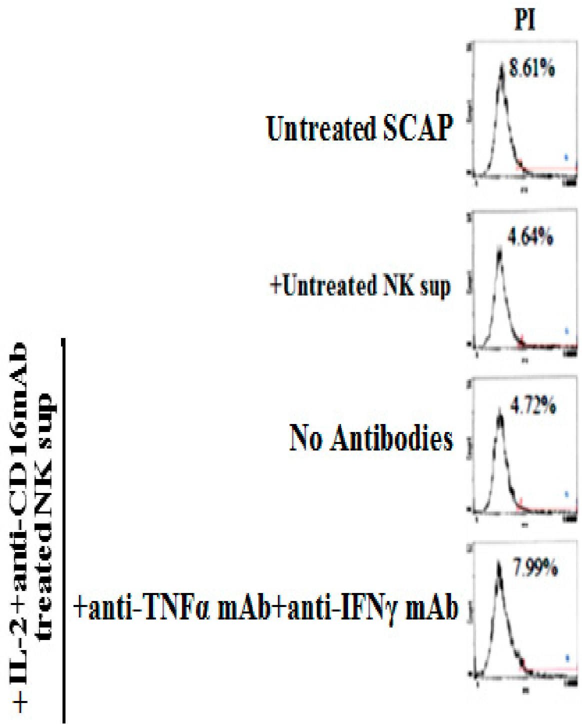
PI
Untreated SCAP
8.61%
+Untreated NK sup
4.64%
No Antibodies
4.72%
+anti-TNFα mAb+anti-IFNγ mAb
7.99%
+IL-2+anti-CD16mAb treated NK sup

C

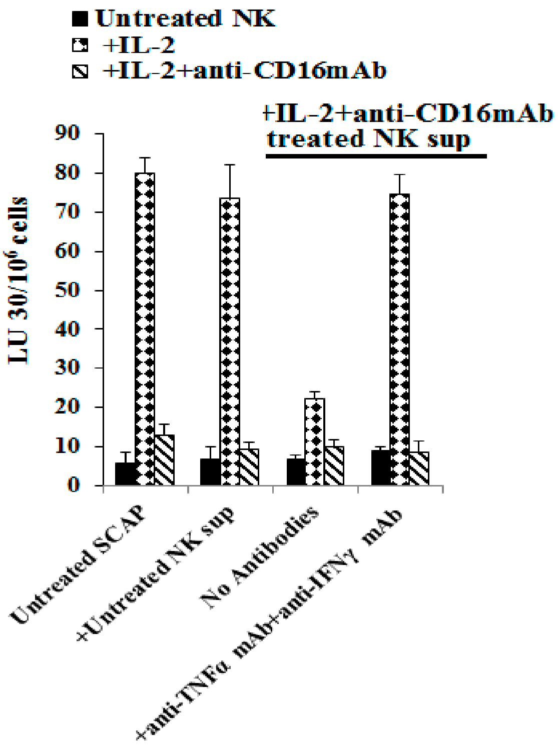
Untreated NK
+IL-2
+IL-2+anti-CD16mAb
+IL-2+anti-CD16mAb treated NK sup
LU 30/10^6 cells
90
80
70
60
50
40
30
20
10
0
Untreated SCAP
+Untreated NK sup
No Antibodies
+anti-TNFα mAb+anti-IFNγ mAb

Figure S5. Treatments with anti-IFN-γ and anti-TNF-α blocked rIFN-γ and rTNF-α NK induced differentiation of SCAP cells. NK cells were prepared as described in Fig. S1A. SCAP cells were treated with rTNF-α (20 ng/mL) or rIFN-γ (200U/mL) or rTNF-α (20 ng/mL) + rIFN-γ (200U/mL) or rTNF-α (20 ng/mL) + rIFN-γ (200U/mL) + αTNFα mAbs (1:100) + αIFNγ mAbs (1:100) for 24 hours. SCAP cells were prepared as described in Fig. S2A, after which, surface expression of CD54, MHC-class I, and B7H1 on SCAP were assessed using flow cytometric analysis **(A)**. NK cells were left untreated or treated with IL-2 (1000 U/ml) before they were used as effectors against ^{51}Cr-labeled hA549 cells in 4 hours cytotoxicity assay. Lytic units (LU) 30/10^6 cells were determined as described in Fig. S2C **(B)**.

A

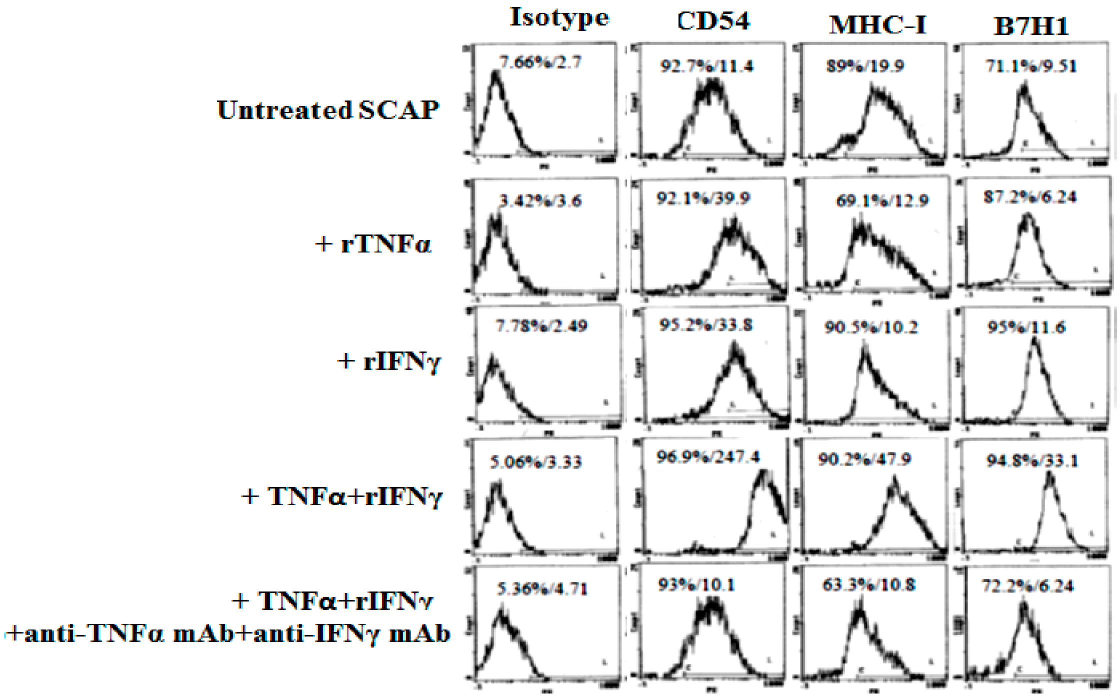

B

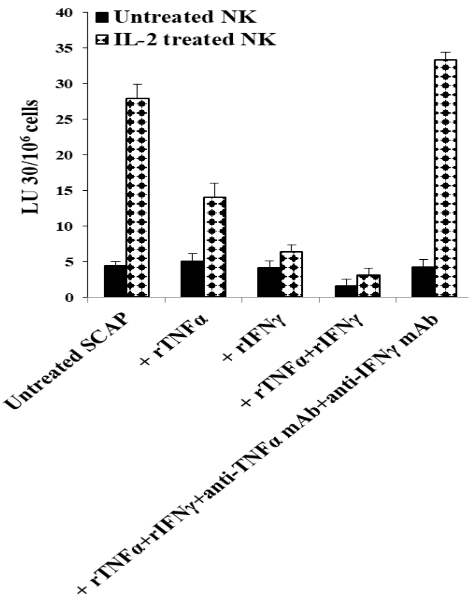

Figure S6. Expression levels of pJNK, pAKT and STAT3 in NK cells and, IFN-γ and TNF-α induced differentiated hA549 NK cells were left untreated or treated with a combination of IL-2 (1000 U/ml) and anti-CD16 mAbs (3μg/mL) overnight before the supernatants were harvested to treat hA549. hA549 cells were treated with rTNF-α (20 ng/mL) or rIFN-γ (200U/mL) or rTNF-α (20 ng/mL) + rIFN-γ (200 U/mL) for 24 hours. hA549 tumors were treated with supernatants of untreated or IL-2 (1000 U/ml) and anti-CD16 mAbs (3μg/mL) treated NK cells alone or with anti-TNFα mAbs (1:100) + anti-IFNγ mAbs (1:100) for six days. After which, the protein was extracted using 200uL of protein lysis buffer and 100uL of protein inhibitor. The presence of pJNK, pAKT and STAT3 was determined using Luminex.

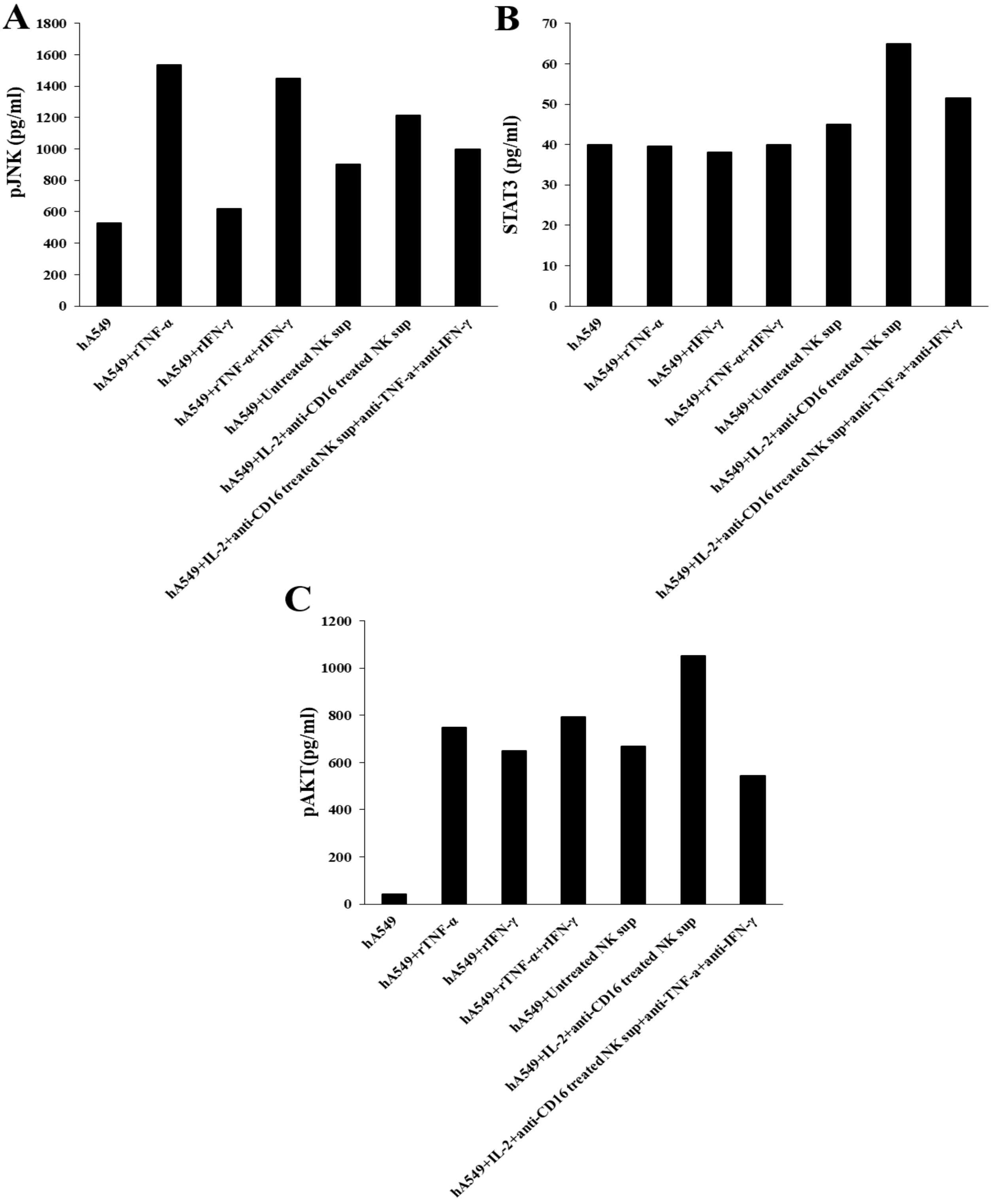

Supplement: Supplementary file 1 [file cells-14-00090-s001.zip › cells-3319023-supplementary.pdf]
